# Variation of the Tensile Properties of Basalt-Fiber-Reinforced Polybutylene Succinate Matrix Composites during Microbial Degradation

**DOI:** 10.3390/polym15071796

**Published:** 2023-04-05

**Authors:** Lovisa Rova, Hiroki Kurita, Shinji Kudo, Sho Hatayama, Teruyoshi Kanno, Alia Gallet--Pandellé, Fumio Narita

**Affiliations:** 1 Department of Frontier Sciences for Advanced Environment, Graduate School of Environmental Studies, Tohoku University, 980-8579 Sendai, Japan; 2 Department of Chemistry, Ångström Laboratory, Disciplinary Domain of Science and Technology, Uppsala University, 75237 Uppsala, Sweden; 3 Department of Ecosystem Studies, School of Environmental Science, The University of Shiga Prefecture, 522-8533 Hikone, Japan; 4 Department of Nutrition, School of Human Cultures, The University of Shiga Prefecture, 522-8533 Hikone, Japan; 5 Department of Materials Science and Engineering, INSA-Lyon, Université de Lyon, F-69621 Villeurbanne, France

**Keywords:** biodegradable, tensile properties, decomposition, polybutylene succinate, basalt fiber

## Abstract

Little is known about how the strength of biodegradable polymers changes during decomposition. This study investigated the changes in the tensile properties of polybutylene succinate (PBS) and basalt-fiber (BF)-reinforced PBS (PBS-BF) composite sheets during degradation in bacterial solutions. Seven days after the start of the experiment, the elongation at break of the PBS specimens decreased significantly, and the PBS-BF composite specimens were characterized by barely any change in ultimate tensile strength (UTS) after immersion in the bacteria-free medium for 7 and 56 days. Meanwhile, when immersed in the bacterial solution, the UTS of the PBS-BF composite specimens showed a tendency to decrease after 7 days. After 56 days, the UTS decreased to about half of its value immediately after fabrication. The degradation of the material was attributed to infiltration of the bacterial solution into structurally weak areas, causing decomposition throughout the material.

## 1. Introduction

One of the most significant environmental challenges that humanity is facing is the problem of plastic pollution. Plastic pollution has been documented in scientific reports since the 1970s; yet, the accumulation of plastic debris in the environment continues to this day [1]. The global production of plastic resins has reached several hundred million metric tons annually, much of which is used in the production of polymer-based composite materials [1,2]. Composite materials have many useful properties, such as their low cost, durability, light weight, and corrosion resistance. However, they are difficult to recycle and the process is not cost-effective. Because of the sheer amount of polymer-based composite materials that are produced, this creates a significant waste problem [1]. Ubiquitous usage contributes to pollution problems when the materials are disposed of in landfills or dumped in natural habitats [1]. Moreover, numerous synthetic additives have been used to reinforce or change the properties of plastic-based materials. Plasticizer leaching, during use or after being improperly discarded in natural environments, raises concerns. Depending on plastics’ chemical makeup, this can harm both wildlife and humans [1]. There is, therefore, a need for new materials that do not pollute the environment (both during fabrication and by the end of use) and that are easier to recycle. In recent years, due to increased environmental concerns, there has been a move toward producing materials based on naturally occurring and renewable resources, as well as recyclable or biodegradable ones. However, bio-based composites often have lower mechanical properties than conventional ones, in which environmental friendliness is not a factor that is much considered [2].

Polybutylene succinate (PBS) is a synthetic and biodegradable semi-crystalline thermoplastic with a melting point of about 115 °C, and mechanical properties and good processability similar to those of commonly used polyolefins, such as polypropylene and polyethylene [3]. The thermal stability and mechanical properties of PBS can be tailored through copolymerization and processing, allowing the polymer to be used in numerous applications such as food packaging or mulch film [4,5]. PBS is synthesized from the two monomers succinic acid and 1,4-butanediol, which are generally produced from petroleum resources [3]. Both monomers can be derived from renewable and bio-based resources, although this approach is more costly and depends on the commercial availability of bio-based 1,4-butanediol [6]. However, considering the increasing oil prices and public demand for more environmentally friendly plastic materials, the bio-based route may become economically advantageous and provide increasing applications for polymers such as PBS [7]. Compared to another commonly used biodegradable polymer, polylactic acid (PLA), PBS is tougher and has a significantly larger elongation at break [3]. Besides its good mechanical properties, one of the most notable properties of PBS is its good biodegradability. Numerous studies have reported the biodegradation of PBS in compost, soil, activated sludge, water, and enzymatic solutions [3,6,8,9]. The demand for PBS is expected to increase in the future as the range of PBS applications is expanded by the development of new composite materials that will reinforce PBS with different properties. For example, Nanni et al. [10] used solid waste generated from wine production, named wine lees, and mixed this in different percentages (10, 20, and 40 phr) with PBS by twin-screw extrusion to obtain a bio-based composite material. The tensile modulus, as well as the creep resistance of PBS, was improved by the addition of the filler material. Shaiju et al. [9] used layered double hydroxides as fillers for PBS, which increased its rate of decomposition when exposed to seawater. The investigators considered the increased rate to be due to the catalytic action of the Mg and Al ions inside the PBS matrix.

One efficient way to improve the mechanical strength of resins such as PBS is to reinforce the polymer matrix by using fibers to produce composite materials. Fiber-reinforced polymers have the advantage of strength combined with lightness, but they are traditionally fabricated using glass and carbon fibers, which are not recyclable [8]. In comparison, natural fibers such as flax, hemp, kenaf, and sisal are cheaper, lighter, and have lesser environmental impacts. These fibers are already being used in combination with polypropylene to construct components in the automotive industry [8]. Zhao et al. [11] synthesized an interesting structural composite by making caterpillar-like continuous hierarchical fibers from bathroom tissue paper, which were then surface treated with poly(dopamine) and γ-methacryloxypropyl trimethoxy silane to improve the interfacial bonding. The fibers were combined with PBS to produce a paper-based biocomposite that had excellent mechanical properties. Another promising reinforcement material based on plant fibers are cellulose nanofibers, which have received increased attention in recent years, with many studies published [12,13,14]. However, compared to inorganic fibers, cellulose and plant-based fibers have the disadvantage of low thermal resistance and sensitivity to ultraviolet light and moisture [8]. Frollini et al. [15] produced a composite based on PBS and reinforced with curaua fibers. They noted that the mechanical properties of PBS were significantly improved by addition of the fibers, and also that water uptake was increased. Water uptake can negatively impact the strength of the composite but also increase the speed of biodegradation, so this can be both a disadvantage and a benefit depending on the context. 

One naturally occurring material that is receiving increasing interest is basalt fiber (BF). This inorganic material is obtained by extrusion molding of volcanic rock with a high basalt content. The process is simple and energy efficient: the basalt rock is crushed, melted, and forced through tiny orifices, forming thin filaments that are collected on a roller [16,17]. As the process is cost-efficient and requires no additives, the fabrication of BFs is significantly cheaper than the fabrication of carbon fibers. Another major benefit of using BFs is the significantly lower environmental impact compared to traditional synthetic fibers because of the simplicity and less intense use of resources during manufacturing [16,17,18]. BFs do not suffer from the same weaknesses to heat and ultraviolet light as many plant-based fibers do; however, they are not fundamentally biodegradable. Nonetheless, they are considered natural because no artificial additives are used, and the basalt rocks that are used can be found in nature virtually anywhere in the world [17]. Lopresto et al. [16] reported that, compared with the more widely used glass-fiber-reinforced laminates, laminates reinforced with BFs performed better in terms of their Young’s modulus, compressive and bending strength, and absorption of impact energy. This was attributed to the good adhesion between the surface of the BFs and the polymer matrix. Li et al. [19] reinforced PBS with 5–20 wt.% BFs and reported a significant increase in tensile strength and modulus with only a few percent of BF loading. With 20 wt.% BFs, the tensile modulus of PBS increased by 490% compared to neat PBS, which was attributed to the good interfacial interactions between BFs and the PBS matrix. They also reported improved thermal stability of the composite material compared to neat PBS. Shen et al. [20] manufactured a composite material by reinforcing cellulose acetate (CA) with BFs. They found that the ultimate tensile strength (UTS) of the material increased by a factor of four compared to pure CA and by a factor of seven when they added a surface-active agent to improve the fiber/matrix adhesion. Compared to the widely used glass fibers, which are susceptible to damage in alkaline conditions, BFs display good thermal and chemical resistance, and they are non-toxic [17,18]. In recent years, Gao et al. [21] investigated the potential of using BFs as reinforcements for concrete to capitalize on the good mechanical properties and the low environmental impact of BFs compared to glass, carbon, polyethylene, or steel fibers. When comparing the environmental impact of BFs to other reinforcing materials, BFs are obtained from natural resources and are biologically inert [19]. This means that although they are not biodegradable, they can be easily recycled by thermal treatment. For these reasons, there is real potential for BFs to replace traditionally used glass and carbon fibers in a variety of applications, bringing with them several environmental benefits. BF-reinforced composites have been extensively studied but PBS has been little investigated as a biodegradable matrix candidate for basalt-fiber-based composites [19].

In the present study, PBS was reinforced with BFs, and the mechanical properties of the composite were evaluated. To test the biodegradability of the material, PBS and the new composite were subjected to degradation in bacterial solution and also in a “blank” solution that did not contain bacteria. Biodegradation testing was performed by immersing PBS and basalt-fiber-reinforced PBS (PBS–BF) composite sheets in a solution containing microorganisms that have been reported to biodegrade PBS [22] for 8 weeks (56 days). To the authors’ knowledge, the change in mechanical properties during biodegradation of composite materials is not widely studied. To understand how biodegradation influences the strength of the material, specimens were removed from the solution periodically to evaluate the changes in mechanical properties by performing tensile tests on the partly degraded specimens. The goal of this study was to improve the understanding of the behavior of BF-reinforced PBS by evaluating the change in mechanical properties during long-term degradation.

## 2. Materials and Methods

### 2.1. Specimen Preparation

**Figure 1:**
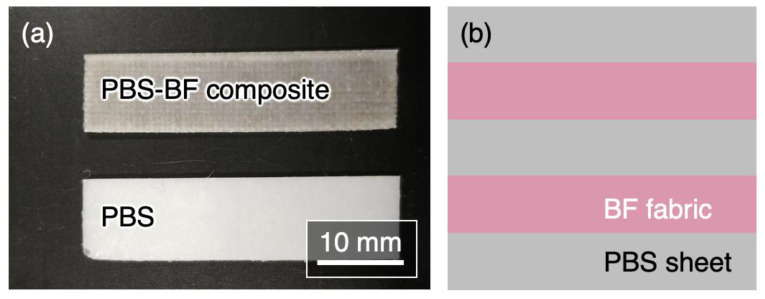
(**a**) Appearance of the PBS–BF composite and the PBS sheets and (**b**) schematic illustration of PBS/BF/PBS/BF/PBS cross-sectional structure.

PBS pellets (Bionolle^®^, Showa Denko KK, Tokyo, Japan) and BF fabric (BWP-108, Zhejiang GBF Basalt Fiber Co., Ltd., Dongyang, China) were used as starting materials. The BF fabric was a plain weave, 0.15 mm thick, with a fiber density of 15 fibers/10 mm for both the warp and weft yarns. The PBS pellets were hot-pressed at 160 °C under 3 MPa uniaxial pressure for 1 min to produce 0.15 mm thick PBS sheets. Two BF fabrics were interleaved between three PBS sheets (50 × 10 × 0.75 mm^3^), forming a PBS/BF/PBS/BF/PBS structure (see Figure 1). For comparison, PBS sheets of the same thickness were prepared under the same conditions. Finally, the PBS–BF composite and the PBS sheets were cut into rectangles of dimensions 50 × 10 × 0.75 mm^3^.

### 2.2. Microbial Degradation Test

**Figure 2:**
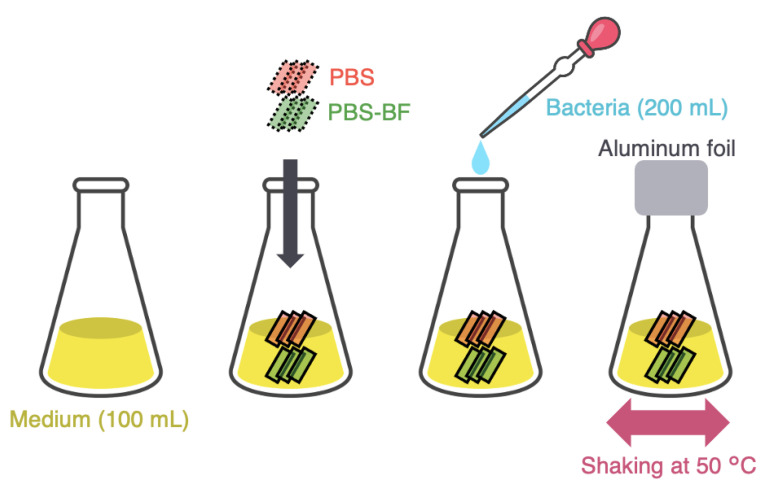
Schematic illustration of the method used in the microbial degradation test of PBS and PBS–BF composites.

Figure 2 summarizes the microbial degradation experiments conducted with the PBS sheets and PBS–BF composite. To prevent contamination, the glassware was heat-sterilized, and the material specimens were sterilized using a sodium hypochlorite solution before they were rinsed with a sterile saline solution (0.85% NaCl). *Microbispora rosea* subsp. *aerata* strain ATCC 27098 was obtained from the American Type Culture Collection (ATCC). This microbial strain was selected because a previous study [22] reported that this bacterial strain degrades PBS. The bacteria were grown in Tryptone Yeast Extract Broth [ISP Medium No. 1 (ISP1); tryptone 5 g/L, yeast extract 3 g/L] at 50 °C for 1 week. The cultured bacterial cells were then transferred into fresh ISP1 and cultivated at 50 °C for an additional week. After this entire cultivation process, the bacterial cells were collected by centrifugation at 12,000 rpm for 3 min, washed by deionized water three times, and re-suspended in sterile saline solution. To normalize the bacterial cells’ number, the absorbance of the prepared bacterial suspension was adjusted to 0.5 ± 0.05 at 600 nm.

Three PBS and three PBS–BF composite specimens were added to triangular flasks containing 100 mL of ISP1 and 200 μL of the adjusted bacterial suspension. As references, additional flasks were prepared without the addition of bacteria, creating PBS and PBS–BF blank specimens. The flasks were sealed with aluminum foil and incubated at 50 °C with a stirring speed of 200 rpm. The test specimens were incubated for a total of 8 weeks (56 days), replacing the ISP1 containing the bacterial solution every 2 weeks. After 1, 2, 4, 6, and 8 weeks of incubation, three specimens of PBS and PBS–BF composites were withdrawn from the flasks, sterilized with a sodium hypochlorite solution for 10 s, and rinsed with a sterile saline solution. These specimens were then used for evaluation of the tensile properties.

### 2.3. Tensile Test

Three specimens each of PBS and PBS-BF composite were prepared for tensile testing. The tensile properties of the PBS and PBS–BF specimens were evaluated at room temperature using a universal testing machine (AG-X plus, Shimadzu Corporation, Kyoto, Japan) with a crosshead velocity of 1 mm/min. Aluminum tabs (15 × 10 × 1 mm^3^) were attached at four locations on the surfaces of the specimens with cyanoacrylate adhesive (Aron Alpha^®^, Toagosei Co., Ltd., Tokyo, Japan) and inserted between the specimens and the jigs to prevent specimen slippage during the tensile tests. The reference length for the calculation of strain was assumed to be the distance between the tabs of 20 mm. Strain gauges were not used. As PBS was expected to highly elongate, extensometers were not used either. Therefore, the fracture elongation of PBS was estimated from the displacement of the crosshead of the universal testing machine. The fracture elongation of the PBS-BF specimens was also estimated from the displacement of the universal testing machine in the same way. The fracture surfaces of the PBS and PBS–BF composite specimens were observed using a field emission scanning electron microscope (FE–SEM; SU-70, Hitachi High-Tech Co., Ltd., Tokyo, Japan) with platinum sputtering on the fracture surface. 

## 3. Results and Discussion

**Figure 3:**
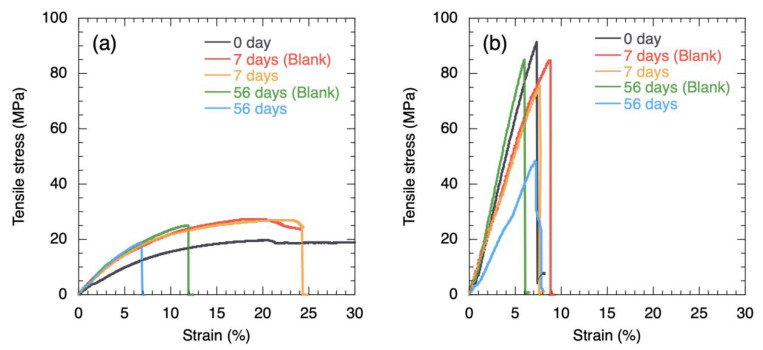
Stress–strain curves for (**a**) PBS and (**b**) PBS–BF composite specimens 7 and 56 days after the start of the decomposition test. Blank refers to specimens immersed in the ISP1 without addition of bacteria.

Although experimental failures reduced the number of tests for some specimens, the test results remained stable, with good reproducibility. Figure 3 shows the stress–strain curves for the PBS and PBS–BF composite specimens. Regarding the mechanical properties of the PBS-BF composites, the addition of BFs to PBS resulted in an increase in UTS by a factor of more than four, but a significant decrease in elongation at break. Immediately after fabrication, the PBS specimens exhibited a fracture elongation of approximately 230% (not shown in Figure 3a). Seven days after the start of the biodegradation experiment, the UTS of the PBS specimens increased slightly, while their fracture elongation decreased significantly. Since there was no significant difference in the tensile properties of PBS specimens immersed in the ISP1 with/without bacteria, the decrease in elongation at break of the polymer was attributed to water absorption and hydrolytic degradation of the PBS specimens in the solution at 50 °C. Due to elevated temperature and humidity, the molar mass is assumed to decrease by chain scission of the ester bonds on the polymer backbone, resulting in embrittlement of the material [23]. After 8 weeks (56 days), the fracture elongation of the PBS specimens decreased significantly while UTS remained largely unchanged. In the medium containing bacteria, the decrease in mechanical properties was exacerbated compared to the one without bacteria, confirming the ability of *Microbispora rosea* subsp. *aerata* to degrade PBS.

**Figure 4:**
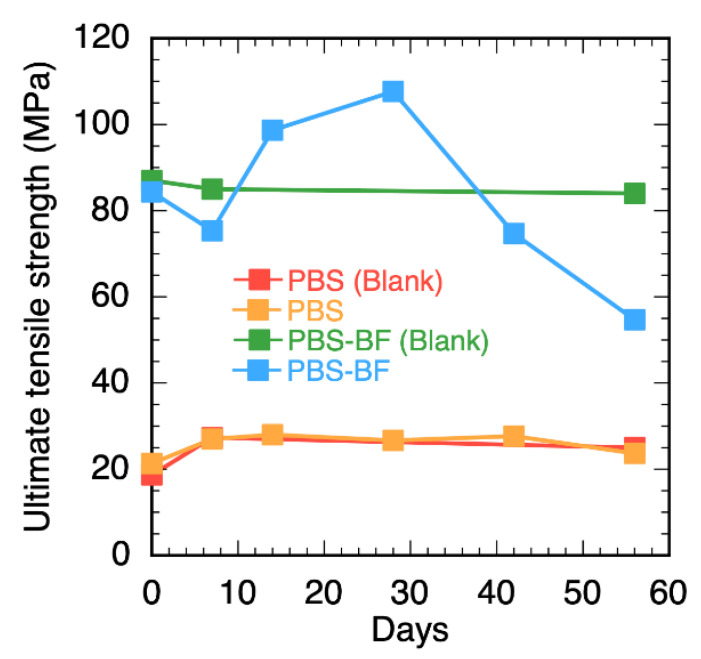
Relationship between the UTS and the time elapsed during the decomposition tests of the PBS and PBS–BF composite specimens.

Figure 4 shows the relationship between the UTS and the time of immersion in the test medium for both PBS and the PBS–BF composite specimens. The UTS of the PBS–BF composite specimens did not change after 56 days when immersed in the medium without bacteria. Meanwhile, when immersed in the bacterial solution for 56 days, the UTS of the PBS–BF composite specimens was reduced to almost half of its original value immediately after fabrication. This proves that the bacteria played a part in degrading the material, and that the degradation was not exclusively due to hydrolysis.

The change in mechanical properties of PBS-based composites during degradation has been little studied. The loss of mechanical properties of the composites in this study could have been caused by both hydrolytic and enzymatic biodegradation of the PBS matrix. It is well known that, in the case of semi-crystalline polymers, the amorphous regions are degraded first, resulting in an increase in crystallinity [24]. Hydrolysis proceeds with cleavage of ester bonds of the polymer chains by water. The hydrolysis rate is higher in amorphous regions as the water molecules can penetrate more easily [24]. A previous study [22] also reported that bacteria selectively degrade amorphous regions. Due to the initial increase in crystallinity at the start of the degradation process when the amorphous regions are degraded, the UTS of the material increases as the polymer becomes more brittle. However, as degradation progresses further, the UTS decreases.

Table 1 compares the UTS for PBS–BF, PLA/PBS–wood flour [25], and PLA/PBS–rice husk [26] composites before and after the biodegradation process. The UTS values of pure PBS before the decomposition process are also shown. The BFs act as a reinforcing material for PBS, while maintaining biodegradability properties similar to other green PLA/PBS composite materials.

**Figure 5:**
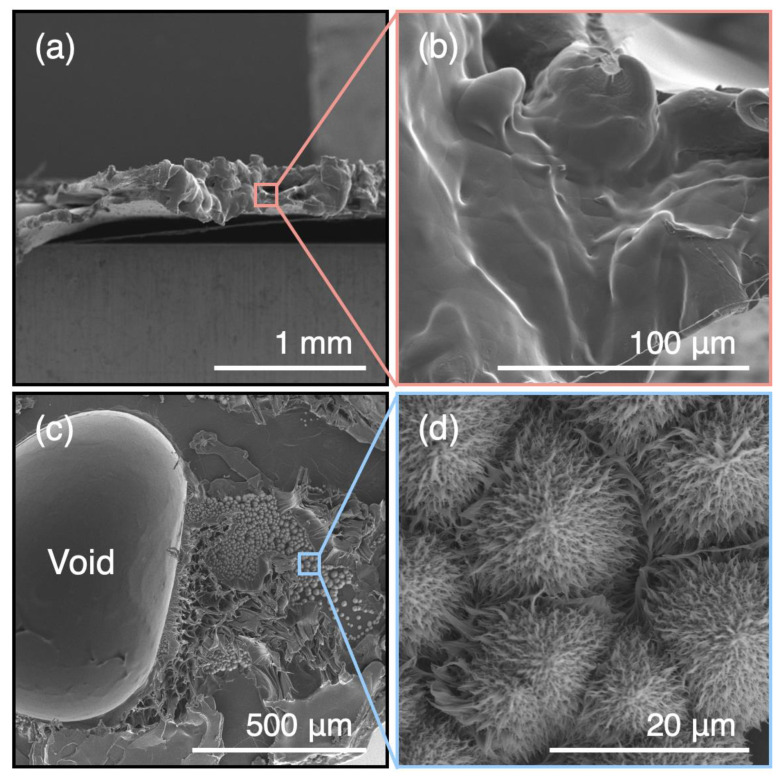
FE–SEM images of the fracture surface of PBS specimens (**a**,**b**) after fabrication and (**c**,**d**) 56 days after the start of the decomposition test.

Figure 5 shows the FE–SEM images of the fracture surfaces of PBS specimens immediately after fabrication and 56 days after the start of the decomposition test. The fracture surfaces of the PBS specimens after fabrication and after tensile testing were similar to those of typical ductile polymers (Figure 5a,b). Meanwhile, the fracture surfaces of the PBS specimens 56 days after the start of the decomposition test showed smooth surfaces (upper part of Figure 5c), typical of brittle materials, and complex biological patterns (Figure 5d). Such patterns were not observed on the fracture surfaces of the PBS specimens after fabrication. However, they were detected on the PBS specimens immersed in the media both with and without bacteria for 56 days, suggesting that they are, at least in part, an effect of hydrolysis. It was impossible to say to what degree—if any—these patterns had been caused by bacterial decomposition. However, the observation of these patterns, both near the surfaces and the center of the PBS tensile specimens 56 days after the start of the degradation test, suggests that the bacterial solution had penetrated the structurally weak areas, causing decomposition to progress throughout the entire material.

**Figure 6:**
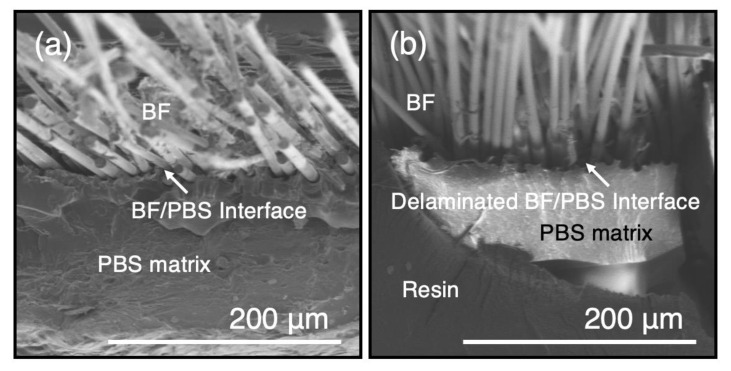
FE–SEM observations of the BF/PBS interface at the fracture surface of the PBS–BF composite specimens (**a**) after fabrication and (**b**) 56 days after the start of the decomposition test.

Figure 6 shows FE–SEM observations of the BF/PBS interface at the fracture surface of the PBS–BF composite specimens immediately after fabrication and 56 days after the start of the decomposition test. Many biological patterns were observed on the fracture surfaces of the PBS–BF composite specimens 56 days after the start of the decomposition test. The delamination at the BF/PBS interface was much larger in the case of the PBS–BF specimens immersed in the bacterial solution (Figure 6b). This result suggests that the bacterial solution had penetrated the BF/PBS interface, which was in physical contact but without chemical bonding, more rapidly and accelerated the biodegradation of the PBS–BF composite specimens. Furthermore, since efficient load transfer from the matrix to the reinforcing fibers is essential for strength development in fiber-reinforced composites such as PBS–BF composites, damage to this interface may have caused the significant decrease in UTS of the PBS–BF composite specimens in the degradation experiments. In addition, a previous study [27] has already reported that BF has high chemical resistance in saline, alkaline, and deionized water solution; subsequently, the loss of its property can be considered negligible. Expecting the PBS matrix to be further degraded during a longer immersion time, while the BFs remain intact, suggests that the fibers could be recovered and recycled. The usual recycling method for inorganic fiber-based composites is by thermal means: the composite is incinerated at high temperature, the polymer matrix burns off, and the fibers can be reclaimed for reuse [28]. However, when using a completely biodegradable matrix, recovery of the non-biodegradable fibers becomes possible in an energy- and cost-efficient way. If the matrix material is fully degraded, recovery of the fibers is simple and prevents potential thermal degradation of the fibers. Persico et al. [29] presented a chemical method that could be used in recycling of BFs: submerging the composite material in a weak acid. Because BFs are chemically inert, they are separated from the matrix material while the mechanical properties of the fibers are kept to a greater degree compared to the thermal recycling method. If biodegradation of the matrix has not progressed fully, removal of the matrix material could be completed using this method.

## 4. Conclusions

In this study, PBS was successfully reinforced with BFs to produce a composite material that could be biologically degraded and/or recycled. The mechanical properties of the composite were evaluated, and the changes in the tensile properties of neat PBS and the PBS–BF composite sheets during decomposition in a bacterial solution were investigated. The produced composite had a UTS of 88 MPa. Seven days after the start of the biodegradation experiment, the fracture elongation of the PBS specimens decreased significantly. These results were attributed not to biodegradation but to water absorption and hydrolytic degradation of the PBS specimens since there was no difference in the tensile properties of the PBS specimens immersed in the media with/without bacteria. Fifty-six days after the start of the experiment, the UTS of the PBS specimens had hardly decreased, but the elongation at break had decreased significantly. The PBS–BF composite specimens were characterized by barely any change in UTS when immersed in the medium without bacteria for 7 and 56 days. Meanwhile, when immersed in the bacterial solution, the UTS of the PBS–BF composite specimens tended to decrease after 7 days; after 56 days, the UTS of the PBS–BF composite specimens decreased to about half of that immediately after fabrication. The deterioration was attributed to infiltration of the bacterial solution into structurally weak areas, causing decomposition throughout the material and significantly decreasing the UTS of the PBS–BF composite specimens. In further studies, the biodegradation of the PBS-BF composite in different media such as soil or compost should be investigated. Furthermore, the recycling process of the BFs is of great interest, and the possibility of retrieving and practically reusing the fibers should be confirmed. To conclude, the PBS-BF composite shows great potential as an environmentally friendly material that is biodegradable and has excellent mechanical properties.

## Figures and Tables

**Table 1:** Comparison of UTS of several PBS matrix-based biocomposites before and after biodegradation testing.

Composite Materials	Test Duration (Days)	UTS (MPa)			Source
		PBS [Before Test]	Composite [Before Test]	Composite [After Test]	
PBS–BF	56	20	86	54	This study
PLA/PBS–wood flour	90	40	50	45	[25]
PLA/PBS–rice husks	180	35	22	12	[26]

## Data Availability

The data presented in this study are available on request from the corresponding author.
